# Exploring the law of color presentation of double-sided heterochromatic digital printing for textile

**DOI:** 10.3389/fpsyg.2022.956748

**Published:** 2022-10-18

**Authors:** Yiting Duan, Min Zhang, Jiu Zhou, Hua Zhou, Liming Xu, Heng Yang

**Affiliations:** ^1^College of Textile Science and Engineering, International Silk Institute, Zhejiang Sci-Tech University, Hangzhou, China; ^2^Hangzhou Honghua Digital Technology Co., Ltd., Hangzhou, China; ^3^School of Design, University of Leeds, Leeds, United Kingdom

**Keywords:** double-sided heterochromatic digital printing, textile digital printing, subjective color difference, objective color difference, regression model

## Abstract

With the upgrading of production technology and design aesthetic of textile products, the development and application of double-sided digital printing technology have been gradually proposed in recent years. The production of double-sided heterochromatic digital printing requires high accuracy for color management, as in the production process, different input devices, display devices, and output devices each have varying color processing capabilities and color performance characteristics, which leads to the transfer of color between different devices and not being accurately reproduced. However, there is little research involved in the color formation laws in the design and production of double-sided heterochromatic digital printing. The purpose of this paper is to explore the prediction model of color presentation in double-sided heterochromatic digital printing. Due to the influence of the fabric thickness, the gap between the textile structure, and the infiltration rate of the printing pigments, the color of each side in double-sided, heterochromatic printing results often differs from the designed color. In this paper, taking chiffon fabric, which is one of the thinnest and has the strongest permeability in silk fabric, as an example fabric base, 24 colors from six hue angles and four chromas were selected as experimental colors. According to the color gamut of the digital printing machine, 15 out of 24 colors were selected as experimental colors. These 15 colors were printed on two sides in pairs to generate 225 color pairs as double-sided experimental samples. For these experimental samples, this paper conducts experiments from both subjective and objective aspects through the combination of subjective evaluation of psychophysics experiments and objective instrument measurement. Through the analysis of experimental data, the color difference prediction under the subjective model (R^2^ = 0.74) and objective model (R^2^ = 0.85) are given, respectively. The dominant color prediction model (R^2^ = 0.75) during double-sided heterochromatic digital printing has also been built. The combination of the three models can predict the color regularity of double-sided heterochromatic digital printing on silk, which may have a certain significance for the design and development of silk double-sided heterochromatic digital printing products.

## Introduction

As people's living standard improves, customers' assessment of textiles is expanding to include not only the texture of the textile material, but also the need for its color. Color is a vital component in human visual information transmission, since it not only delivers the designer's message but also reflects people's feelings and has a positive influence on their spirit and conduct (Ou et al., 2012). The color of textile patterns has become one of the most essential variables for customers to consider when buying textiles, particularly among young consumers who are fashion savvy and individualistic. According to the research, more than 40% of customers' first consideration when purchasing textiles is color. If the preferred textiles do not have their favorite color, they will opt to temporarily not buy or buy others instead, as it is usually not easy for people to give up their favorite colors (Wang et al., 2016). In American marketing, there is a 7-s rule that states that customers will determine their buying intention within 7 s, and color subjective accounts for 67% of the decision element in such a short period (Qin, 2014). This demonstrates the significance of color in consumer behavior, and the influence of textile color in affecting product look is even more intangible.

To fulfill the need for manufacturing and design, textile digital printing technology, a complete high technology combining computer, electronic information, machinery, and other disciplines, was developed in the 1990's (Yang et al., 2010). Digital printing technology is a significant achievement in the world of textile printing, and it is the result of societal development and current trends. Digital printing is the result of the organic combination of printing technology and computer technology, and is based on computer graphics, digital manufacturing, and computer networks. Image information is input into the computer, edited, and calculated by the color separation system, and then the dye is directly sprayed onto the fabric or media by the printing system to produce beautiful printing patterns (Tyler, 2005). On the one hand, the problem with the current situation of digital printing color management in China is that the technology of digital printing color management is primarily imitated from the paper printing industry, whereas digital printing of textiles is very dissimilar from paper printing, whether it is hardware equipment, ink, fabric, or process, and textile digital printing is much more complex than paper printing (Gooby, 2020). As a result, the advancement of color management technology in the printing sector needs to be paid attention, but it cannot be entirely replicated. Rather, the peculiarities of digital textile printing need to be enhanced and expanded. Another issue is that color difference is at the heart of textile quality, and the role of color difference measurement in the process of textile quality evaluation is becoming abundantly obvious (Gangakhedkar, 2010), but there is a scarcity of literature research and systematic application of double-sided printing color difference detection about silk fabric. There has been limited research, in particular, on color location and color creation criteria in color design and production in double-sided heterochromatic digital printing. Because of the thinness of the fabric, gaps between the tissue structure, and the permeability of the printing pigments, the printed color in double-sided digital printing often differs from the planned hue angle (Tyler, 2011). Color differences occur in all color reproduction sectors, and this phenomenon is most noticeable in chiffon textiles, thus the purpose of this research is to explore the color pattern of double-sided heterochromatic digital printing with high permeability. The experimental fabric used in this research is a high-permeability chiffon fabric. This research uses a combination of the subjective evaluation method of psychophysics experiment and instrumental measurement method to conduct the experiment from both subjective and objective perspectives to look at the problem of color difference in chiffon fabric when doing double-sided heterochromatic digital printing. The three models combined can forecast the color presentation pattern of double-sided heterochromatic digital printing on silk, which may be useful in the design and development of silk double-sided heterochromatic digital printing goods.

## Materials and methods

**Figure 1:**
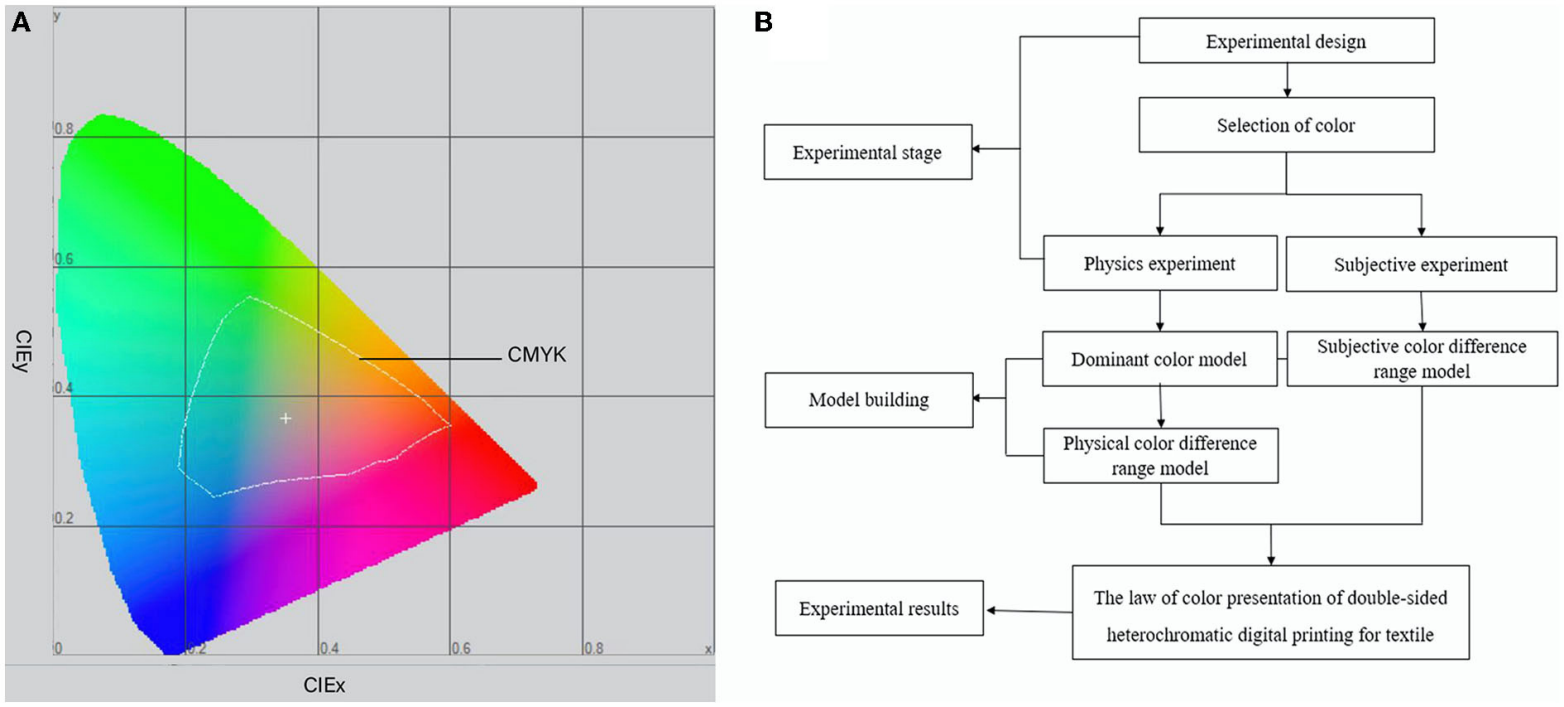
**(A)** Color gamut range of ICC profiles. **(B)** Experimental flow chart.

This paper primarily uses a combination of objective measurement and subjective measurement to analyze the color presentation pattern of chiffon fabric when doing double-sided heterochromatic digital printing, and it provides a prediction model of color difference size based on subjective and objective measurements. In order to make the color printed by the digital printer to be closer to the designed color, the design colors were color managed by using the ICC profile of the digital printing machine provided by Hangzhou Honghua Digital Technology Co., Ltd., and the color gamut range of this ICC is shown in Figure 1A. A total of 210 colors from the digital printing machine's color range were chosen as experimental samples, and objective measurement and subjective measurement studies were performed on these experimental samples. The findings of 30 individuals assessing the degree of color difference between 210 single- and double-sided experimental samples under the visual influence solely were gathered in the subjective experiment. In the objective experiments, a CM700d spectrophotometer was used to measure the L^*^a^*^b^*^ chromaticity values of the front and back sides of the experimental samples, and the color difference values (hereinafter referred to as ΔE^*^) of the corresponding colors of the single- and double-sided experimental samples are calculated using the CIEDE2000 color difference formula based on the CIELAB color space. Finally, this paper analyzes the causes for the color difference of the experimental samples in subjective and objective assessments, as well as the connection of color difference size in subjective and objective evaluations. In this method, the color presentation prediction model during double-sided heterochromatic digital printing of textiles is determined. Figure 1B depicts the experimental flow.

### The choice of color

**Figure 2:**
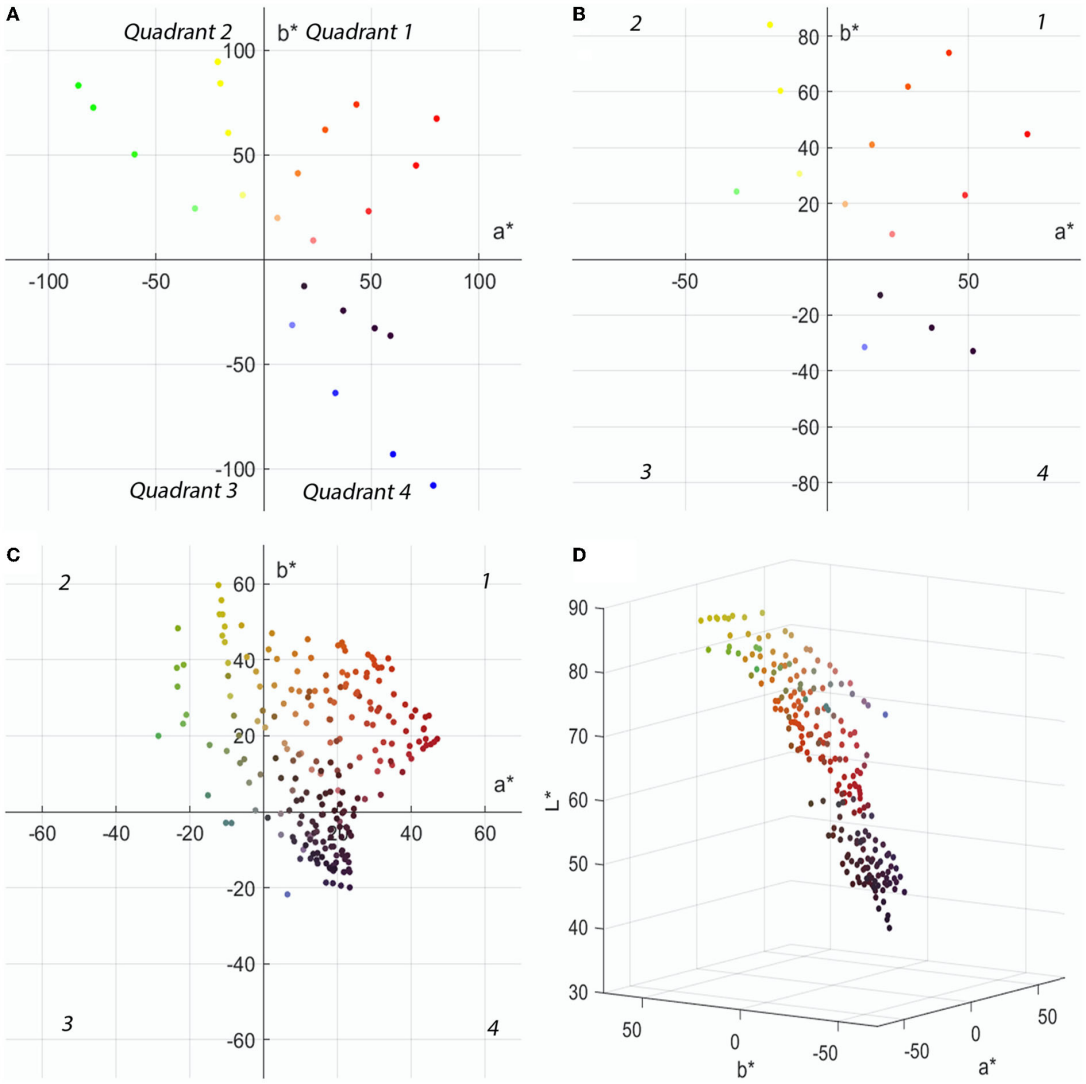
The color distribution in CIELAB space: **(A)** Two-dimensional distribution of 24 colors in CIELAB space. **(B)** Two-dimensional distribution of 15 colors in CIELAB space. **(C)** Two-dimensional distribution of 210 colors in CIELAB space. **(D)** Three-dimensional distribution of 210 colors in CIELAB space.

In this paper, 24 colors are chosen from six hue angles and four color chroma, and the colors are distributed in CIELAB color space with the equal lightness of a^*^-b^*^ two-dimensional distribution, as shown in Figure 2A, in one, two, and four quadrants, each with eight colors, and the 24 colors are grouped in pairs (two colors in each group) to generate a total of 576 different colors. In order to ensure the authenticity and validity of the data, the specimen (single-sided chiffon color card) required for the experiment was folded in five layers according to the measurement method of textile color (Gangakhedkar, 2010), and the L^*^a^*^b^*^ value of the specimen was measured by CM700d spectrophotometer (the specular component is SCI) in the case of the fabric sample without light transmission. It was discovered that there is a significant disparity between the chromaticity value and the intended color. Given that the color of digital printing is mixed by four CMYK colors, and that digital printing is used to control the output color by computer, color management was performed in accordance with the ICC profile of the digital printing machine. Some colors were discovered to be beyond the color gamut of the printer, resulting in severe color differences. After removing the super gamut colors, 15 colors remained, and their chromaticity values L^*^a^*^b^*^ are shown in Supplementary Table 1, and their two-dimensional distribution in CIELAB color space with equal lightness a^*^-b^*^ is displayed in Figure 2B. They are positioned in one, two, and four quadrants, with 7 colors in the first quadrant and 4 colors in each of the second and fourth quadrants, and these 15 colors are paired in groups to produce 225 distinct color samples; among them, 210 colors are heterochromatic. Figure 2C depicts the a^*^-b^*^ two-dimensional distribution of these 210 color samples with equal lightness in CIELAB color space, with |a^*^| and |b^*^| values in the (0, 70) interval; in the fourth quadrant, the |a^*^| values are in the (0, 30) interval, and the |b^*^| values are in the (0, 40) interval, which is red-blue with lower saturation. As shown in Figure 2D, most of the yellow samples have the highest lightness value between 70 and 90; green has the second highest lightness value, which is between 60 and 80; red lightness values are between 30 and 70, which demonstrate a larger interval; the lightness values of blue and purple samples are relatively low, yet they are more than 30. Due to the limitations related to the experimental material and the duration of the experiment, only 210 color samples were evaluated in this paper.

### Materials and methods

Psychophysics experimental method and instrument objective measurement method are primarily employed to subjectively and objectively analyze the color presentation pattern of chiffon fabrics in conducting double-sided heterochromatic digital printing, while providing a prediction model of the level of color difference under subjective and objective measurements.

#### Objective color experiment method

**Figure 3:**
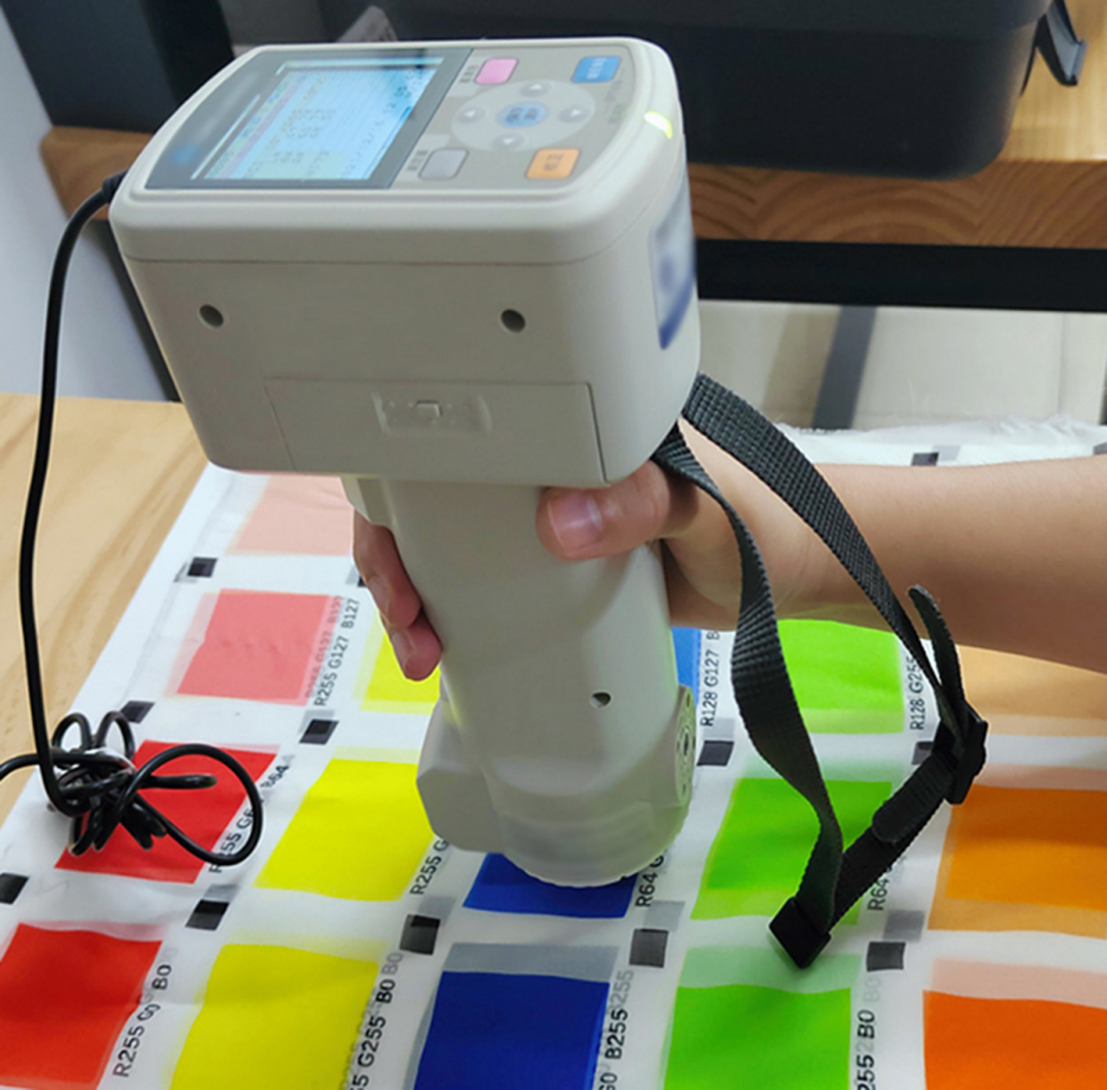
Physical experiment diagram.

The objective color assessment approach in this research is to use a CM700d spectrophotometer to evaluate the chromaticity values L^*^a^*^b^*^ of 210 colors based on CIELAB color space, as shown in Figure 3. The samples are measured in five layers to ensure that the samples are opaque; all samples were in a consistent environment and placed on the 90% reflective white plate for measurement. Before measuring, the spectrophotometer was set to D65 light source, d/8 (diffuse lighting 8° light receiving), specular inclusion (SCI), measuring aperture of 8 mm, and used a 10-degree CIE standard observer. In addition, the repeatability of Konica Minolta CM700d was ΔE^*^ab ≤ 0.04 and the inter-instrument variation was ΔE^*^ab ≤ 0.2, after which the white board and black board were calibrated, and the specimens were measured with the L^*^, a^*^, and b^*^ values of each color card recorded. The objective color difference (hereinafter referred to as ΔE^*^) of the experimental samples was calculated using the standard color difference method CIEDE2000 in CIELAB space (Luo et al., 2001; Wang et al., 2012; Westland and Pan, 2017). The relationship between the objective color difference and the color's lightness, chroma, and hue angle was then investigated.

#### Subjective experimental method

**Figure 4:**
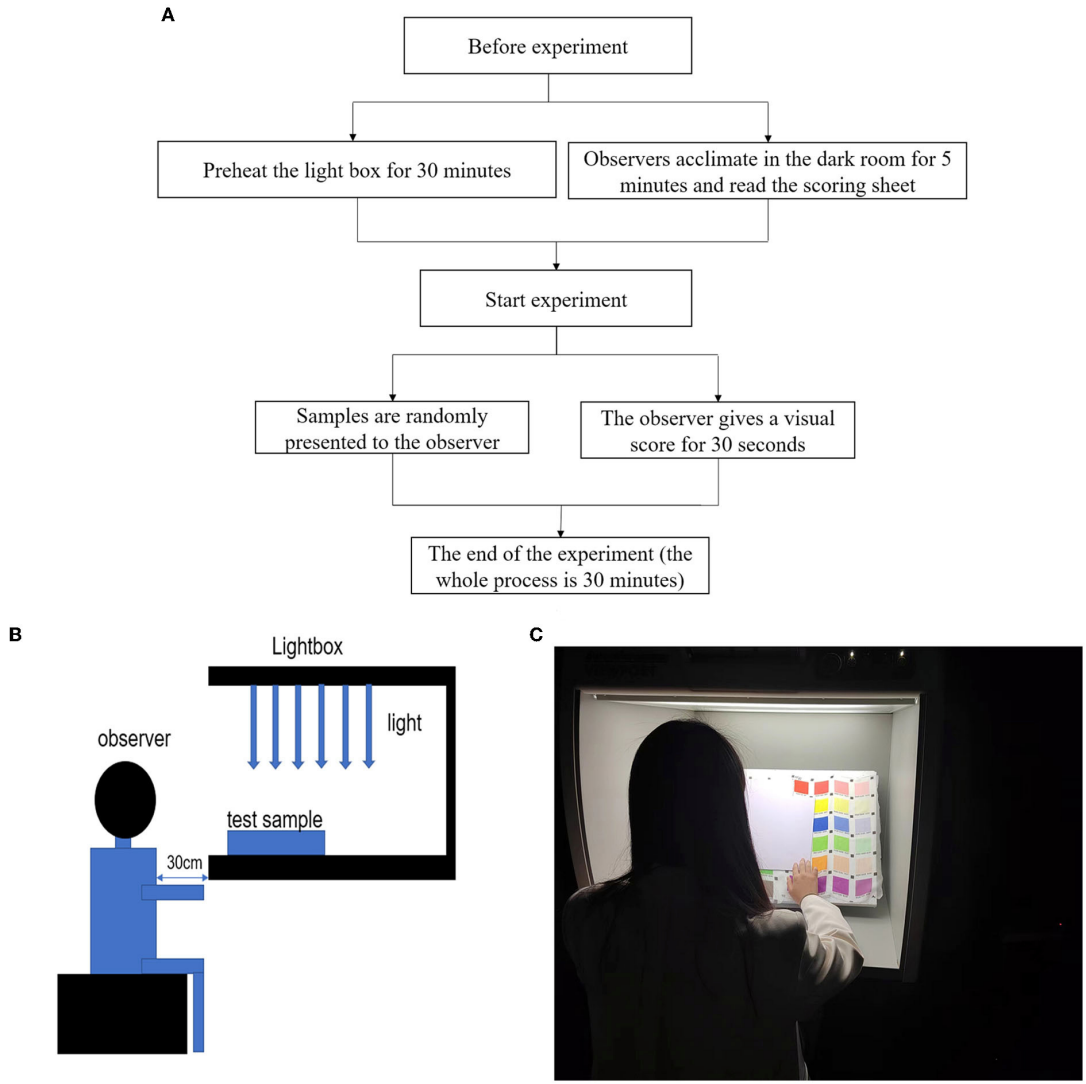
**(A)** Subjective experiment flow chart, **(B)** The experiment environment, and **(C)** Experiment procedure.

The assessment of the quality of textile digital printing needs to be based on people's feelings, and the major and most accurate evaluation of this feeling is people's subjective evaluation. As a result, this subjective experiment estimates the extent of the color difference based on people's visual ratings. The experimental sample of double-sided heterochromatic digital printing was subjected to a visual experiment in order to investigate the relationship between the subjective perceptible color difference, color lightness, and color phase when doing double-sided heterochromatic digital printing on semi-transparent chiffon fabric. The sample size for the research was computed using G^*^power 3.1 software (Faul et al., 2007) with a medium effect size of 0.50, and the optimal sample size for the paired-sample *t*-test was 34 groups under statistical test power of 1–β = 0.80 and α = 0.05. This study includes 30 undergraduate and postgraduate students aged 20–25 years, all of whom have similar study background, and equal numbers of men and women were submitted to a repeat experiment after 1 week, and a total of 60 sets of experimental data were obtained. According to the Ishihara color blindness test, none of the 30 volunteers chosen for the experiment were color defiant. The experimental steps are shown in Figure 4A.

This test was conducted in a dark room with no other lighting source, the light source was D65, the illumination of the light box used in the experiment was 2250 under a D65 light source, and the light box was painted in standard gray (L^*^ of 50). The observer is required to maintain an upright sitting position while staring directly at the specimens in the light box, as indicated in Figures 4B,C. The observer (subjective evaluator) visually compares the 210 experimental samples of double-sided heterochromatic digital printing to the single-sided printed specimens using the scoring criteria, and assigns evaluation scores based on the magnitude of the scores to judge the size of the printed color difference. Ten color similarity score ranges were created to assess the double-sided heterochromatic digital printed textiles. Table 1 shows the specifics of the scores.

**Table 1:** Perception experiment scoring rules.

**Grade**	**Score interval**	**Evaluation standard**
1	[0.0–1.0]	There is almost no color difference
2	[1.1–3.0]	There is a slight color difference
3	[3.1–5.0]	There is obvious color difference
4	[5.1–7.0]	There is a very large color difference
5	[7.1–10.0]	Different color from standard

At the same time, the test sample (double-sided heterochromatic digital printing) and standard sample (single-sided digital printing) color cards were put in a lightbox with a D65 light source (only one color was shown at a time, and when one color block was shown, the other color blocks were blocked with white cardboard). Before beginning the experiment, the light source D65 in the light box was switched on at full lightness for 30 min to warm up. Before entering the dark room, observers read the experimental procedures and scoring criteria and waited for 5 min to adjust to the dark environment before doing the visual experiment individually. The observer is required to tell the size of the color difference between the samples within 5 s, and the time between consecutive samples can be adjusted according to the observer's own state. The color difference of the samples in this experiment is completely subjectively determined by the observer and is not subject to external interference. For example, if the color between the sample and reference pairs was the same, the response would be “0”; if the color difference between the subject sample and the standard sample was different, the response would be a bigger number ranging from 0.1 to 10. The answer was recorded by the recorder and could not be corrected. One week later, the observers were asked to repeat the experiment. The specimens were given to the observer in random order for each observation to prevent the influence of the observer's memory effect on the experimental outcomes.

## Results analysis of the objective color difference of double-sided heterochromatic digital printing

When double-sided digital printing was delivered, Pearson correlation analysis was performed between the color difference values and the lightness, chroma, and hue of the color, as well as the model of the dominant color and the model of the color difference range.

### Objective color difference law of double-sided heterochromatic digital printing

In this paper, the objective color difference ΔE^*^ (color difference between the single-sided control group and double-sided experiment) and L^*^C${}_{\text{ab}}^{*}$ h _ab_ were calculated using the CIEDE2000 formula in order to investigate the relationship between objective color difference and color lightness, chroma, and hue angle during double-sided heterochromatic digital printing, and the results of Pearson correlation analysis are presented in Table 2.

**Table 2:** Pearson correlation between ΔE ^*^and L^*^, C${}_{\text{ab}}^{*}$, and h_ab_.

	L*	C*_ab_	**h_ab_**
Pearson correlation coefficient	−0.30**	−0.32**	0.07
Sig.	0.000	0.000	0.314

**Indicates that the test method used in this paper is a two-tailed significance test method.

Pearson's correlation between ΔE^*^, L^*^, and C${}_{\text{ab}}^{*}$ was found to be significant, Sig. = 0.000 ≤ 0.001. There was no substantial Pearson association between ΔE^*^ and h_ab_. The Pearson correlation coefficients of ΔE^*^ with L^*^ and C${}_{\text{ab}}^{*}$ were −0.3 and −0.32, respectively, indicating a negative relationship between ΔE^*^, lightness, and chroma, i.e., the smaller the lightness and chroma of the designed color within a certain range, the larger the resulting single- and double-sided color difference. To summarize, the objective color difference between single- and double-sided digital printing is largely impacted by lightness and chroma.

### Determination of the dominant color during double-sided heterochromatic digital printing

Because chiffon is one of the thinnest silk textiles, the color presentation will be impacted by the color of the opposite side when undertaking double-sided heterochromatic digital printing. This paper examines the relationship between the color difference between the front and back sides of the double-sided experimental samples and the difference in L^*^C^*^h between the front and back sides to determine which colors dominate the presentation of fabric color when two different colors are matched to do double-sided heterochromatic digital printing. The difference of color difference between the front and back of the experimental sample is recorded as ΔE^*^_f − b_. When ΔE^*^_f − b_ < 0, it means that the objective color difference between the front side of the experimental sample and the designed color, ΔE${}_{\text{f}}^{*}$, is smaller than the ΔE${}_{\text{b}}^{*}$. That is, at this time, the color presentation is more biased toward the color of the front of the experimental sample, and the front color is the dominant color. The difference in the L^*^C^*^h of the front and back specimens are recorded as ΔL^*^_f − b_, ΔC${}_{\text{b}\left(\text{f}-\text{b}\right)}^{*}$, and Δh _ab(f − b)_. However, in the color system of L^*^C^*^h, when calculating the hue difference, it is generally expressed as ΔH${}_{\text{ab}}^{*}$ (Wang and Dou, 2005), and the Pearson correlation test was done between ΔL^*^_(f − b)_, ΔC${}_{\text{ab}\left(\text{f}-\text{b}\right),}^{*}$ and ΔH${}_{\text{ab}}^{*}$_(f − b)_, as shown in Table 3. The results of Pearson correlation analysis showed that ΔE${}_{\left(\text{f}-\text{b}\right)}^{*}$ was positively correlated with ΔL${}_{\left(\text{f}-\text{b}\right)}^{*}$ and negatively correlated with ΔC${}_{\text{ab}\left(\text{f}-\text{b}\right)}^{*}$. That is, the larger ΔL${}_{\left(\text{f}-\text{b}\right)}^{*}$, the larger ΔE${}_{\left(\text{f}-\text{b}\right)}^{*}$, the larger the ΔC${}_{\text{ab}\left(\text{f}-\text{b}\right)}^{*}$, and the smaller the ΔE${}_{\left(\text{f}-\text{b}\right)}^{*}$.

**Table 3:** Pearson correlation between ΔE^*^_f − b_ andΔL^*^_f − b_, ΔC ${}_{\text{f}-\text{b}}^{*}$, and ΔH${}_{\text{ab}}^{*}$_(f − b)_.

	ΔL*_f − b_	ΔC *_f − b_	ΔH*_ab__(f − b)_
Pearson correlation coefficient	0.80**	−0.60**	−0.01
Sig.	0.000	0.00	0.991

**Indicates that the test method used in this paper is a two-tailed significance test method.

Through the Pearson correlation, it is feasible to determine how to perform double-sided heterochromatic digital printing while the dominant color and the lightness difference between the front and back side are closely related, in order to investigate whether the dominant color presentation law can be expressed as Δ L^*^_f − b_, ΔC^*^_f − b_, and ΔH${}_{\text{ab}}^{*}$_(f − b)_. There is a linear relationship between ΔL^*^_f − b_, ΔC^*^_f − b_, and ΔH${}_{\text{ab}}^{*}$_(f − b)_, according to the regression analysis, and the regression equation can be represented as follows:

#### Dominant color determination


(1) $$\begin{array}{l} △\text{E }*{\;}_{\left(\text{ f}-\text{b}\right)}=\;-0.102+4.554△\text{L }*{\;}_{\;(\text{f}-\text{b})}-1.\text{084}\\ \;△\text{C }*{\;}_{\text{ab}(\text{ f}-\text{b})}+0.024△\text{H }*{\;}_{\text{ ab}(\text{f-b})}({\text{R}}^{2}=0.745) \end{array}$$

**Figure 5:**
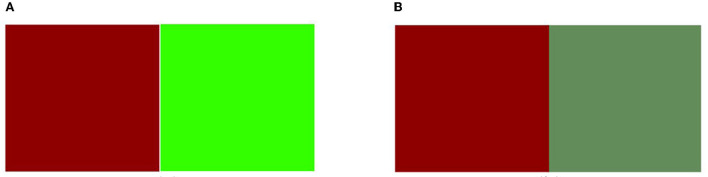
**(A)** Contrasting color pairs of the same chroma. **(B)** Contrasting color pairs of the same lightness.

Where ΔL^*^_d − s_ is the lightness difference between the front and back, ΔC ${}_{\left(\text{d}-\text{s}\right)}^{*}$ is the chroma difference between the front and back, and ΔH${}_{\text{ab}}^{*}$_(f − b)_ is the hue difference between the front and back. Significance analysis R^2^ = 0.75 suggests that the model fits well and may be used to forecast the dominant color in double-sided heterochromatic digital printing. The results show that, when double-sided heterochromatic digital printing is applied to chiffon fabric, the color with the lowest lightness is the dominant color. When the lightness is fixed, the chroma influences the color pattern, and the color with the highest chroma being the dominant color. Furthermore, compared with lightness and chroma, the influence on hue is low according to determining the front and back color difference. As shown in Figure 5A, when the red and green (lightness of the green is higher than the red) are paired in groups to conduct double-sided heterochromatic digital printing, the color presented more in favor of the red. When the lower chroma green and higher chroma red are paired to generate two-sided heterochromatic digital printing, the color is more inclined to the higher chroma red, as illustrated in Figure 5B. When grouping two distinct hue angles, this model might assist the designer in predicting the dominating color.

### The color difference range of double-sided heterochromatic digital printing

The previous section investigated the problem of determining the dominant color when doing double-sided heterochromatic digital printing in chiffon fabric. The following section investigates the color difference between double-sided heterochromatic digital printing and single-sided printing, thereby looking for whether there is some kind of pattern among the color difference rules. The color difference between the single-and double-sided experimental specimens is analyzed based on the difference between the L${}_{\text{ab}}^{*}$ C${}_{\text{ab}}^{*}$ h _ab_ values of single-and double-sided colors. At the same time, the color difference between single- and double-sided experimental fabrics are calculated using the CIEDE2000 formula, is recorded as ΔE^*^, and the difference between the L${}_{\text{ab}}^{*}$ C${}_{\text{ab}}^{*}$ h _ab_ values of single- and double-sided specimens are recorded as ΔL${}_{\left(\text{d}-\text{s}\right)}^{*}$, ΔC${}_{\text{ab}\left(\text{d}-\text{s}\right)}^{*}$, and ΔH${}_{\text{ab}}^{*}$_(d − s)_, while the Pearson correlation test is carried out between ΔE${}_{\left(\text{d}-\text{s}\right)}^{*}$ and ΔL^*^_(d − s)_, ΔC${}_{\text{ab}\left(\text{d}-\text{s}\right)}^{*}$, and ΔH${}_{\text{ab}}^{*}$_(d − s)_, as shown in Table 4. The results of Pearson correlation analysis showed that ΔE^*^_(d − s)_ was negatively correlated with ΔL^*^_(d − s)_ and ΔC${}_{\text{ab}\left(\text{d}-\text{s}\right)}^{*}$, and positively correlated with ΔH${}_{\text{ab}}^{*}$_(d − s)_, and the correlation with ΔL^*^_(d − s)_ was the most significant. That is, within a certain range, the larger the ΔL^*^_(d − s)_ or the ΔC${}_{\text{ab}\left(\text{d}-\text{s}\right)}^{*}$, the smaller the ΔE^*^d-s. The larger the ΔH${}_{\text{ab}}^{*}$_(d − s)_, the larger the ΔE^*^_(d − s)_.

**Table 4:** Pearson correlation between ΔE^*^and ΔL^*^_(d − s)_, ΔC^*^_ab(d − s)_, and ΔH${}_{\text{ab}}^{*}$_(d − s)_.

	ΔL*_(d − s)_	ΔC*_ab(d − s)_	ΔH*_ab__(d − s)_
Pearson correlation coefficient	−0.84**	−0.18**	0.75**
Sig.	0.000	0.000	0.000

**Indicates that the test method used in this paper is a two-tailed significance test method.

Through Pearson correlation, it is possible to determine the close relationship between the color difference of double-sided heterochromatic digital printing and single-sided printing, lightness difference, and chroma difference. In order to investigate whether the law between the objective color difference, the lightness, chroma, and hue angle of the experimental sample can be expressed by ΔL^*^_d − s_, ΔC^*^_d − s_, and ΔH${}_{\text{ab}}^{*}$_(d − s)_, the regression analysis between ΔE^*^_(d − s)_ and ΔL ${}_{\left(\text{d}-\text{s}\right)}^{*}$, ΔC${}_{\text{ab}\left(\text{d}-\text{s}\right)}^{*}$, and ΔH${}_{\text{ab}}^{*}$_(d − s)_ is carried out, and it can be found that there is a linear correlation between them, and the regression equation can be expressed as follows:

#### Objective color difference range


(2) $$\begin{array}{l} \begin{array}{lll} △E^{*}{}_{\left(d-s\right)} & = & 5.884-0.698△L^{*}{}_{\left(d-s\right)}-0.114△C^{*}{}_{ab(d-s)} \end{array}\\ \;\begin{array}{ll} + & 0.497△H_{ab(d-s)}^{*}(R^{2}=\;0.854) \end{array} \end{array}$$

where ΔL^*^_d − s_ is the lightness difference between double-sided and single-sided printing, ΔC ${}_{\text{d}-\text{s}}^{*}$ is the chroma difference between double-sided and single-sided printing, ΔH${}_{\text{ab}}^{*}$_(d − s)_ is the hue angle difference between double-sided and single-sided printing. Significance analysis R^2^ = 0.85 demonstrates that the model fits well and may be used to forecast the color difference range while doing double-sided heterochromatic digital printing. The results indicate that when producing double-sided heterochromatic digital printing on chiffon fabric, there will be a large color difference between the single-sided and the double-sided printing, and the effect is greatly affected by ΔL${}_{\left(\text{d}-\text{s}\right)}^{*}$ and ΔH${}_{\text{ab}\left(\text{f}-\text{b}\right)}^{*}$, and is least affected by ΔC ${}_{\text{ab}\left(\text{d}-\text{s}\right)}^{*}$. In conclusion, the model can predict the degree of color difference between single-sided and double-sided digital printing according to the L^*^C${}_{\text{ab}}^{*}$ h _ab_ value of the specified color.

In order to evaluate the performance of the regression models (1) and (2), six testing colors were selected. The L^*^a^*^b^*^ values of these colors are shown in Supplementary Table 2, and the six colors were paired in pairs to generate a total of 30 heterochromatic double-sided experimental samples. According to the analysis method of the above experiment, the ΔE^*^_(f − b)_ and ΔE^*^_(d − s)_ of the 30 colors are calculated, and the experimental data of these 30 colors are substituted into the above two regression equations, respectively. Then, the Pearson correlations between ΔE^*^_(f − b)_ and ΔE^*^_f − b(regressionprediction)_, and between ΔE^*^_(d − s)_ and ΔE${}_{\text{d}-\text{s}\left(\text{regressionprediction}\right)}^{*}$ were analyzed. The Pearson correlation coefficient between ΔE${}_{\left(\text{f}-\text{b}\right)\text{and}}^{*}$ ΔE^*^_f − b(regressionprediction)_ is 0.50, and the Pearson correlation coefficient between ΔE^*^_(d − s)_ and ΔE${}_{\text{d}-\text{s}\left(\text{regressionprediction}\right)}^{*}$ is 0.90. Therefore, it can be seen that the size of the color difference simulated by the regression equation is closely related to the actual size of the color difference between samples and that both can be used to simulate the actual color difference, among which the simulation performance of the model (2) is better than that of the model (1).

## Analysis of subjective evaluation of double-sided heterochromatic digital printing

In the subjective experiment, the participants were asked to visually score the double-sided heterochromatic digital printing under only visual influence, with the degree of the rating representing the perceived color difference. A Pearson correlation analysis was conducted between visual rating and color lightness, chroma, and hue angle in order to provide a model of subjective color difference range for double-sided digital printing and to discuss the color characteristics within the subjectively acceptable color difference range for double-sided digital printing on chiffon fabric.

### Observer repeatability

**Figure 6:**
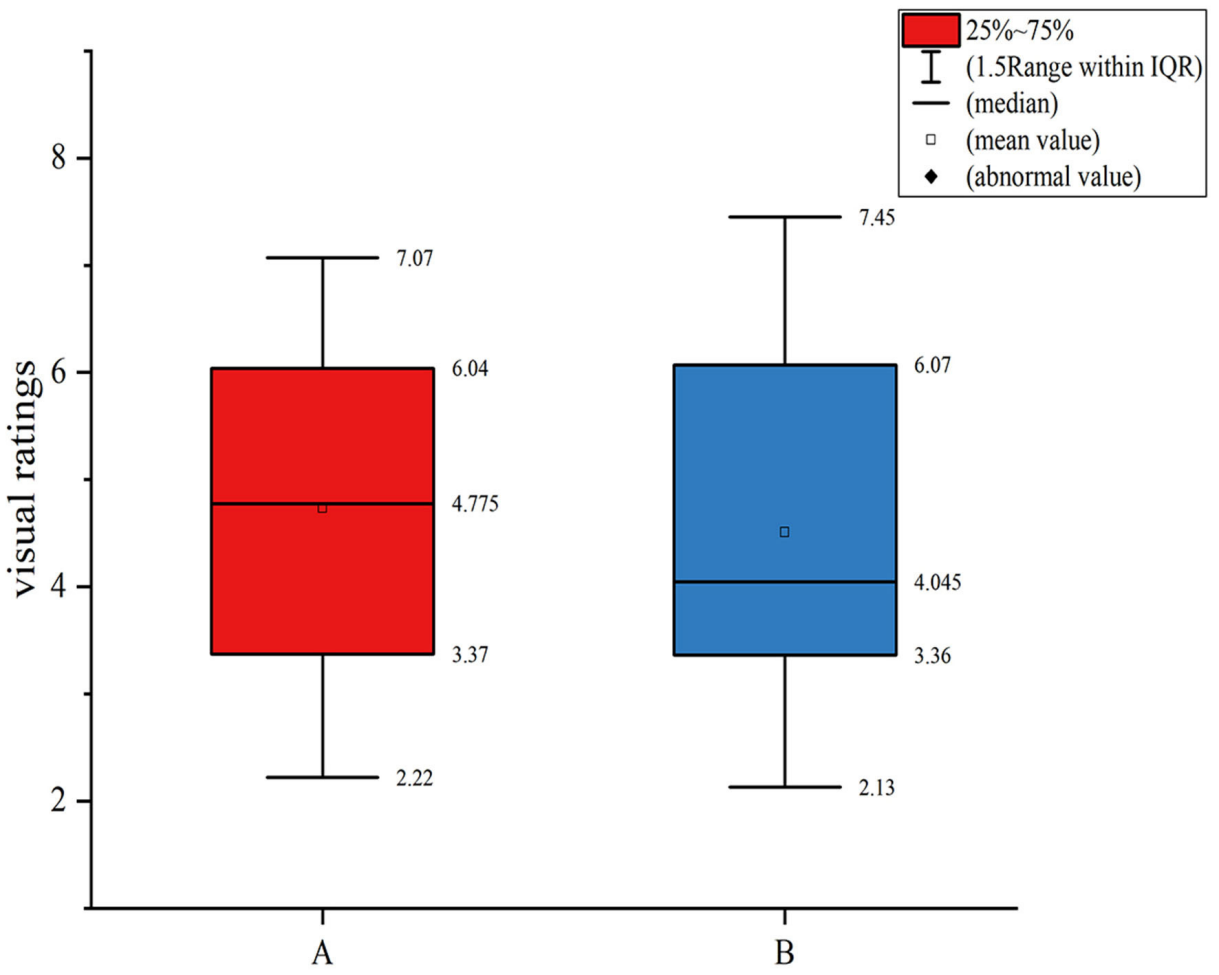
Boxplot of 30 subjects' visual scores.

Before evaluating the validity of the test results, it is necessary to examine the repeatability and precision of the observer test. The main reasons for visual matching errors include visual fatigue caused by long-term observation, psychological factors of observers during the matching process, and fewer repetitions of visual matching experiments. In order to assure the correctness of the assessment test results, the volunteers were instructed to manage their time efficiently so that the subjective experiment could be finished within 30 min. Figure 6 depicts a box plot of the visual ratings of the initial experiment data A and the repeated experiment data B, revealing that there were no outliers in the subjective ratings of the 30 participants, suggesting that the subjective ratings of all 30 subjects were utilized for data analysis.

Two measures were used to examine the reliability of the experimental data: inter-observer variability and intra-observer variability. The former indicates how well the observers agreed with each other in visual responses. For each observer, the inter-observer variability was determined by the root mean square (RMS) value:


(3) $$\begin{array}{l} RMS=\sqrt{\frac{\underset{\text{i}}{\sum }x_{i}-\;\bar{x}}{\text{N}}} \end{array}$$

Here x_i_ represents the visual score for observers “i” to the sample, $\bar{x}$ represents the mean visual score of all observers to the sample, and N is the number of the sample. The lower the RMS value, the more closely the observers agreed with each other. Inter-observer variability, however, is concerned with how repeatable is each observer's response. This was also determined by Equation (3), with $\bar{x}$ being replaced by y_i_, the response of observer “i” at the second occurrence of the sample. To evaluate intra-observer variability, all samples were presented two times for each observer. The lower the RMS value, the more repeatable is the observer's response. Table 5 shows the inter-observer and intra-observer variability for all observers, while the mean value for female observers was lower than for male observers, indicating that female observers were more consistent and repeatable.

**Table 5:** Inter-observer and intra-observer variability for experiment.

**Male**	**Inter-observer** **variability**	**Intra-observer** **variability**	**Female**	**Inter-observer** **variability**	**Intra-observer** **variability**
Observer 1	1.17	1.64	Observer 1	2.18	1.74
Observer 2	2.25	1.38	Observer 2	0.66	1.58
Observer 3	3.29	1.64	Observer 3	1.94	2.08
Observer 4	2.21	1.95	Observer 4	1.36	1.27
Observer 5	2.39	2.84	Observer 5	2.23	3.30
Observer 6	2.42	1.62	Observer 6	2.50	3.56
Observer 7	2.19	1.98	Observer 7	1.56	1.37
Observer 8	1.33	1.85	Observer 8	3.13	2.23
Observer 9	1.74	2.32	Observer 9	1.91	1.95
Observer 10	2.07	1.75	Observer 10	1.33	1.50
Observer 11	1.90	1.93	Observer 11	1.42	1.80
Observer 12	2.53	1.91	Observer 12	2.38	1.68
Observer 13	2.25	1.62	Observer 13	1.72	1.08
Observer 14	1.36	1.64	Observer 14	2.26	1.52
Observer 15	2.05	2.27	Observer 15	1.70	1.36
**Male mean**	2.08	1.89	**Female mean**	1.88	1.87

### Subjective color difference law of double-sided heterochromatic digital printing

Following the analysis of the relationship between objective color difference and chroma, lightness, and hue, Pearson correlation analysis was performed between the subjective scores and L${}_{\text{ab}}^{*}$ C${}_{\text{ab}}^{*}$ h _ab_ values of these 210 experimental samples in order to investigate the relationship between the intensity of subjective color difference, the lightness, and the chroma of color during double-sided heterochromatic digital printing on translucent chiffon fabric and the analysis results as shown in Table 6.

**Table 6:** Pearson correlation between perceptual color difference and L^*^, C${}_{\text{ab}}^{*}$, and h_ab_.

	L*	C*_ab_	**h_ab_**
Pearson correlation coefficient	−0.18**	−0.48**	0.10
Sig.	0.008	0.00	0.145

**Indicates that the test method used in this paper is a two-tailed significance test method.

The findings demonstrate a substantial Pearson correlation between the size of the perceived color difference and the L^*^ and C${}_{\text{ab}}^{*}$ (Sig < 0.01), suggesting that the model is extremely significant. The Pearson correlation with hue angle was not statistically significant. The Pearson correlation coefficients for perceived color difference with L^*^ and C${}_{\text{ab}}^{*}$ were−0.18 and−0.48, respectively, indicating a negative correlation between perceived color difference and lightness and chroma; the smaller the L^*^ and C${}_{\text{ab}}^{*}$ of the designed color within a certain range, the larger the perceived color difference, and in double-sided digital printing, the objective color difference between single- and double-sided digital printing is strongly influenced by lightness and chroma. Meanwhile, using Pearson correlation, a significant association (Sig < 0.01) between subjective color difference and objective color difference (correlation coefficient of 0.84) was found. It shows that the perceived color difference under subjective assessment and the objective color difference under instrumental measurement have a positive connection and are closely associated, with the lower the color difference, the lower the perceived color difference rating. To some extent, it can be considered that when the subjective color difference is < 3, the objective color difference is acceptable. The intensity of objective color difference may be utilized to determine whether the color difference of this sample is subjectively acceptable by humans in practical production applications.

### Subjective color difference range of double-sided heterochromatic digital printing

The prediction model of the color difference range based on the objective measurement of the instrument is presented in the preceding research on the objective color difference range of double-sided heterochromatic digital printing. To determine whether the subjective color difference when doing double-sided heterochromatic digital printing can be directly predicted by the relationship between the lightness, chroma, and hue angle of the color, and whether there is a difference between men and women when making the rating (Zhang et al., 2019), the differences in L^*^C${}_{\text{ab}}^{*}$ h_ab_ between the single- and double-sided experimental samples were denoted as ΔL${}_{\left(\text{d}-\text{s}\right)}^{*}$, ΔC ^*^_ab(d − s)_, and ΔH${}_{\text{ab}\left(\text{d}-\text{s}\right),}^{*}$ and the Pearson correlation analyses were conducted between the mean values of subjective and combined subjective scores of men and women, ΔL${}_{\left(\text{d}-\text{s}\right)}^{*}$, ΔC ^*^_ab(d − s)_, and ΔH${}_{\text{ab}\left(\text{d}-\text{s}\right)}^{*}$, as shown in Table 7. To investigate whether the magnitude of the visual scores was influenced by gender, Pearson correlation analysis was done between the objective color difference ΔE^*^_(d − s)_ (men and women), as well as the combined visual scores under the instrumental measurements, as shown in Table 7. The Pearson correlation analysis results showed that the correlation between the subjective scores as well as the combined subjective scores of men and women and the lightness difference between single- and double-sided printing was significant, with women having a higher correlation than men. In the correlation analysis between subjective ratings and objective color difference for both genders, the correlation between subjective ratings and objective ratings measured by the instrument was also higher for women than for men, and female observers responded more accurately to color than male observers. This finding is consistent with the findings of Yang and Baroun 's team (Baroun and Alansari, 2006; Yang and Li, 2016).

**Table 7:** Pearson correlation coefficient.

	ΔL*_(d − s)_	ΔC *_ab(d − s)_	ΔH*_ab(d − s)_
**Pearson correlation between visual scores of females**			
**and** ΔL*_(d − s)_, Δ**C** *_ab(d − s)_, ΔH*_ab(d − s)_			
Pearson correlation coefficient	−0.79**	−0.02	0.74**
Sig.	0.000	0.764	0.000
**Pearson correlation between visual scores of males**			
**and** ΔL*_(d − s)_, Δ**C** *_ab(d − s)_, ΔH*_ab(d − s)_			
Pearson correlation coefficient	−0.75**	−0.095	0.72**
Sig.	0.000	0.155	0.000
**Pearson correlation between visual scores of comprehensive**			
**visual scores and** ΔL*_(d − s)_, Δ**C** *_ab(d − s)_, ΔH*_ab(d − s)_			
Pearson correlation coefficient	−0.77**	−0.07	0.735**
Sig.	0.000	0.333	0.000
	**Male**	**Female**	**Comprehensive**
**Pearson correlation between physical color difference**			
ΔE*_(d − s)_ **and male, female, comprehensive visual score**			
Pearson correlation coefficient	0.80**	0.86**	0.84**
Sig.	0.000	0.000	0.000

**Indicates that the test method used in this paper is a two-tailed significance test method.

The Pearson correlation shows that in the subjective assessment of double-sided heterochromatic digital printing, there is a close correlation between the perceived color difference (visual rating), the difference in lightness, and the hue between single and double sides. In order to investigate whether the law of the perceived color difference can be expressed by ΔL${}_{\left(\text{d}-\text{s}\right)}^{*}$, ΔC ^*^_ab(d − s)_, and ΔH${}_{\text{ab}\left(\text{d}-\text{s}\right)}^{*}$, the regression analysis of the mean values of objective scores, the composite objective scores of men and women, ΔL${}_{\left(\text{d}-\text{s}\right)}^{*}$, ΔC ^*^_ab(d − s)_, and ΔH${}_{\text{ab}\left(\text{d}-\text{s}\right)}^{*}$ was conducted, which revealed a linear correlation between them, and the regression equation can be expressed as follows:

*Subjective prediction of female visual score*


(4) $$\begin{array}{l} \begin{array}{lll} F & = & 1.375-0.131△L^{*}{}_{\left(d-s\right)}+0.001△C^{*}{}_{ab(d-s)} \end{array}\\ \;\begin{array}{ll} + & 0.117△H^{*}{}_{ab(d-s)}(R^{2}=\;0.754) \end{array} \end{array}$$

*Subjective prediction of male visual score*


(5) $$\begin{array}{l} \begin{array}{lll} M & = & 2.545-0.137△L^{*}{}_{\left(d-s\right)}-0.012△C^{*}{}_{ab(d-s)} \end{array}\\ \;\begin{array}{ll} + & 0.122△H^{*}{}_{ab(d-s)}(R^{2}=\;0.709) \end{array} \end{array}$$

**Figure 7:**
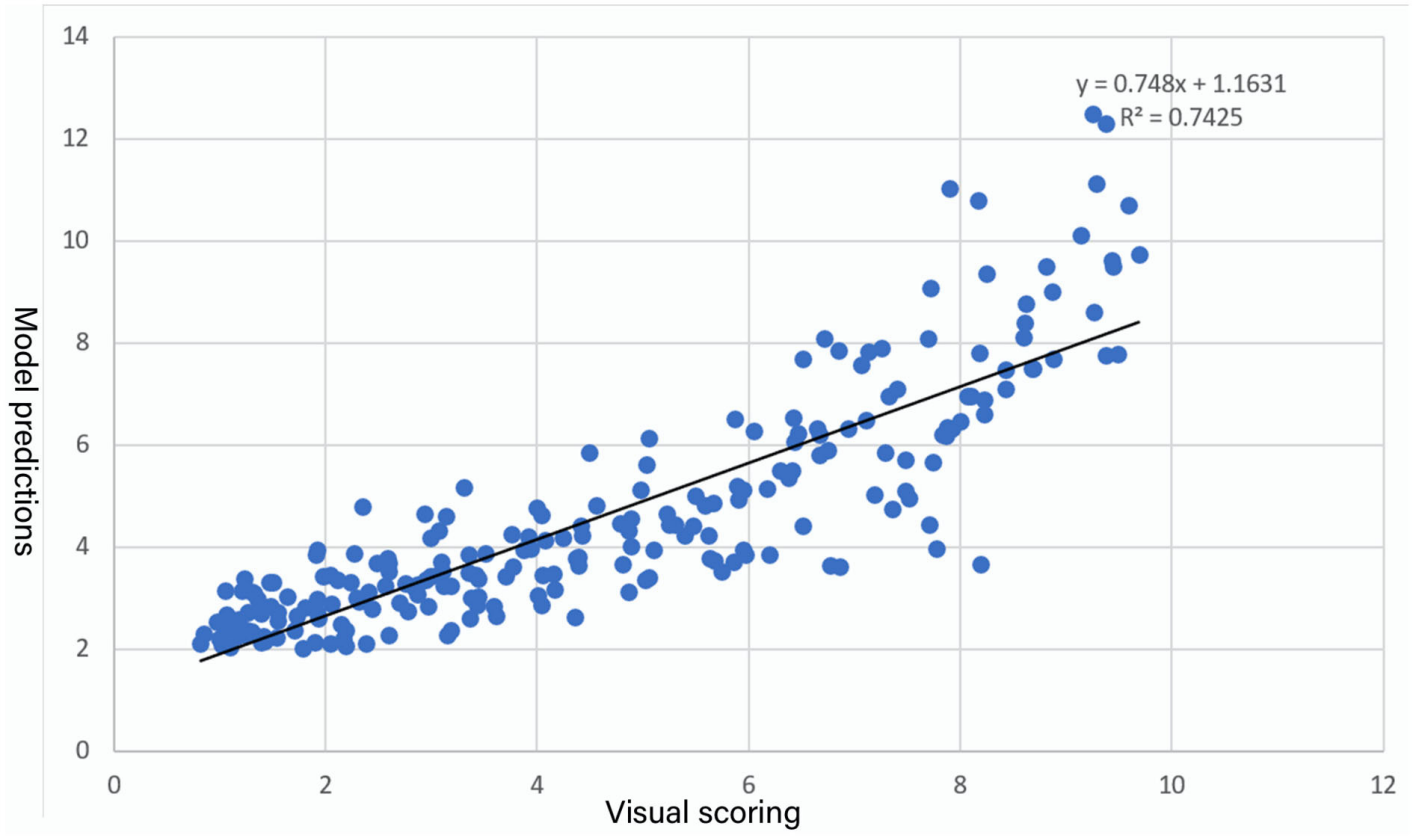
Scatter plot between visual scoring and model predictions.

*Subjective prediction of composite visual score*


(6) $$\begin{array}{l} \begin{array}{lll} C & = & 1.956-0.135△L^{*}{}_{\left(d-s\right)}-0.006△C^{*}{}_{ab(d-s)} \end{array}\\ \;\begin{array}{ll} + & 0.119△H^{*}{}_{ab(d-s)}(R^{2}=0.743) \end{array} \end{array}$$

where ΔL^*^_(d − s)_ is the lightness difference between double-sided and single-sided printing, ΔC ${}_{\left(\text{d}-\text{s}\right)}^{*}$ is the chroma difference between double-sided and single-sided printing, and ΔH${}_{\text{ab}\left(\text{d}-\text{s}\right)}^{*}$ is the hue angle difference between double-sided and single-sided printing. Based on the significance analysis of the regression equation, the regression model fitting degree among female visual scores and ΔL${}_{\left(\text{d}-\text{s}\right)}^{*}$, ΔC ^*^_ab(d − s)_, and ΔH${}_{\text{ab}\left(\text{d}-\text{s}\right)}^{*}$ is the highest (R^2^ = 0.75), and the regression model fitting degree between male visual score and ΔL${}_{\left(\text{d}-\text{s}\right)}^{*}$, ΔC ^*^_ab(d − s)_, as well as ΔH${}_{\text{ab}\left(\text{d}-\text{s}\right)}^{*}$ is the lowest (R^2^ = 0.71), while the regression model fitting degree between combined visual score and ΔL${}_{\left(\text{d}-\text{s}\right)}^{*}$, ΔC ^*^_ab(d − s)_, and ΔH${}_{\text{ab}\left(\text{d}-\text{s}\right)}^{*}$ is R^2^ = 0.74, and a scatter plot between visual results and model predictions is shown in Figure 7.

When executing double-sided heterochromatic digital printing, this model could be used to forecast the range of perceived color differences. When combined with Pearson correlation analysis, it is possible to infer that lightness has the biggest influence on subjective rating in the subjective evaluation, which is consistent with the results of Xiaoming Zhao's research teams (Zhao et al., 2014). From this model, it can be concluded that the subjective color difference is greatly affected by ΔL^*^_(d − s)_ and ΔH${}_{\text{ab}\left(\text{d}-\text{s}\right)}^{*}$, and is less affected by ΔC ^*^_(d − s)_. This conclusion is consistent with the objective color difference model (2), but the subjectively acceptable color difference range is larger than that of the objective color difference. In conclusion, when carrying out the color design of double-sided digital printing of different colors, the lightness difference needs to be considered first, followed by the hue difference.

When provided subjective measures of colors, female observers reacted more correctly to colors than males, according to an analysis of the regression models under the visual evaluations from subjective males and females. Moreover, it was discovered that the combined mean score provided by women was 3.95, and the combined mean score supplied by men was 5.21 by assessing the magnitude of the visual ratings for both genders. This leads to the conclusion that in this trial, women were more tolerant to color than men.

### Color distribution within the range of subjectively acceptable color difference

It is known from the related research that the experimental samples with objective color difference < 3 in textiles are acceptable (Liu et al., 2007). The 210 groups of double-sided heterochromatic digital printing experimental samples selected for this experiment have only nine groups of objective color difference < 3, accounting for 4.2%, proving that when doing double-sided heterochromatic digital printing on chiffon fabric, it will generally produce a large color difference, so there is a limitation in using the above objective experimental results model. The appraisal of the printing quality needs to be based on human feelings, with subjective human judgment being trustworthy. According to the scale of subjective experiment rules, the subjectively acceptable color difference in this experiment is < 3.

**Figure 8:**
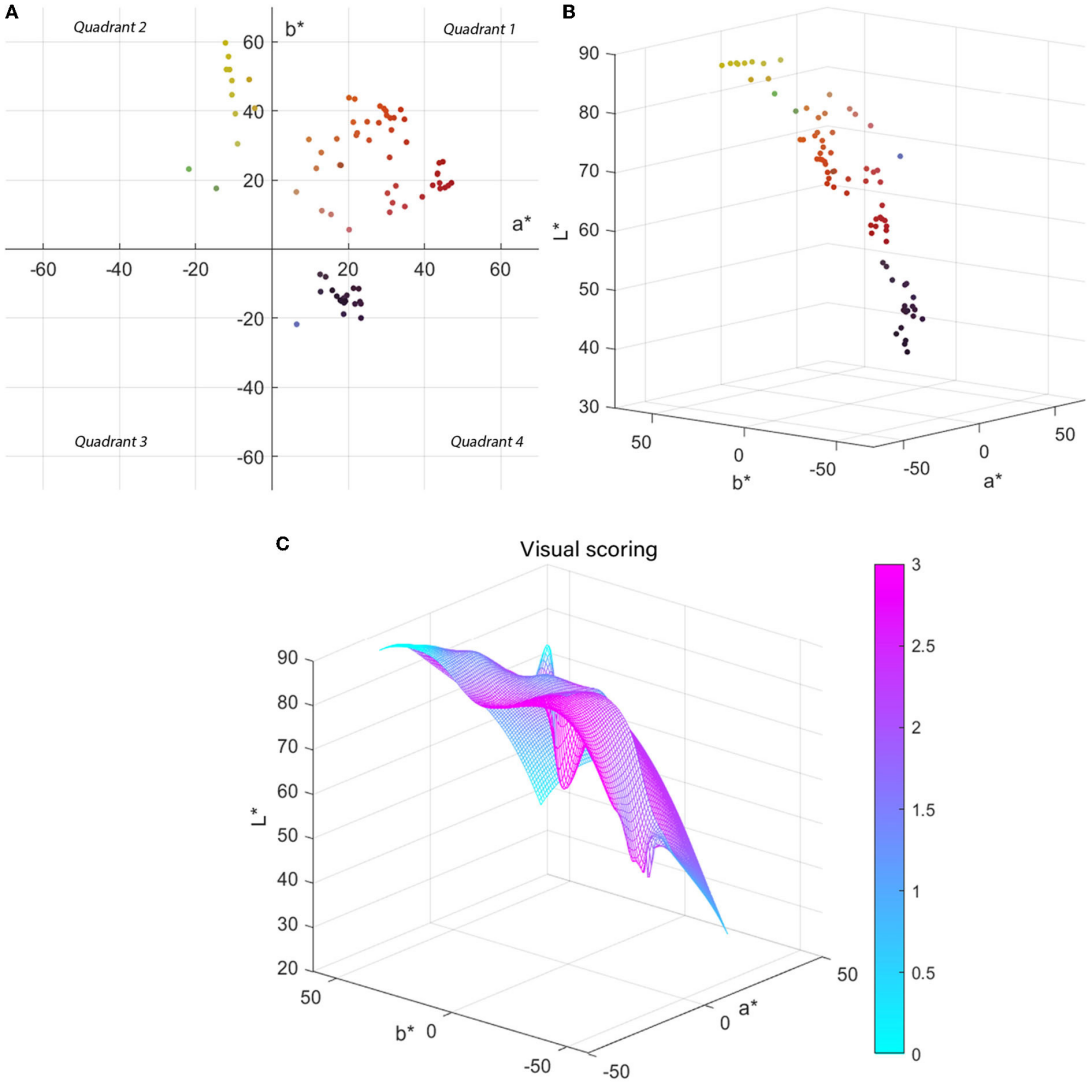
**(A)** Two-dimensional distribution of 78 colors in CIELAB space. **(B)** Three-dimensional distribution of 78 colors in CIELAB space. **(C)** Correlation model of chromaticity value L*a*b* and human visual score.

The subjective color difference of 210 groups of double-sided heterochromatic digital printing experimental samples chosen for this research was < 3, with 78 groups accounting for 37.1%, and the physical color difference at this time is all within 15. It demonstrates that when making double-sided heterochromatic digital printing on chiffon fabric, the range of subjective acceptable color difference to human sight exceeds the actual color difference. To visualize the distribution of chromaticity values of the experimental samples in CIELAB color space, the two-dimensional distribution of a^*^- b^*^ when the color difference is < 3 is shown in Figure 8A. These colors are not distributed in quadrant 3; the values of a^*^ and b^*^ in the first and second quadrants can exceed 50, while the distribution below 20 is less and even close to zero. Values of |a^*^| in the fourth quadrant are in the (10, 30) range, whereas |b^*^| values are in the (20, 40) interval. As demonstrated in Figure 8B for the distribution of 78 colors in CIELAB space, there is no color dispersion near the origin.

This paper modeled the L^*^ and a^*^, b^*^ in MATLAB to more intuitively analyze the color characteristics within the subjective acceptable color difference range during double-sided heterochromatic digital printing, as shown in Figure 8C, with colors indicating the relationship between the L^*^a^*^b^*^ values of the fabric and the subjective visual rating; the colors from blue to pink indicate the deepening of the influence of L^*^a^*^b^*^ values on subjective visual scores.

In conclusion, based on an analysis of the distribution of chromaticity values L^*^a^*^b^*^ in CIELAB color space with a subjective visual score of < 3, when it comes to double-sided heterochromatic digital printing on chiffon fabric, the colors within the subjective acceptable color difference have the following characteristics: when the color is in the first quadrant, the lightness ranges between 55 and 75, and the chromatic value a^*^b^*^ ranges between 15 and 55. When the color is in the second quadrant, the lightness is between 20 and 65, the chromatic value a^*^ is between−15 and 0, and b^*^, which appears to be bigger, is between 20 and 60. When the color is in the fourth quadrant, the chromatic value is between 30 and 45, and the chromatic value a^*^b^*^ is between 10 and 25.

## Conclusion and prospects

### Conclusion

Based on the combination of subjective evaluation of psychophysics experiment and objective instrument measurement, the main goal of this paper is to analyze the relationship between the subjective color difference and objective color difference and lightness, chroma, and hue of the color when it comes to double-sided heterochromatic digital printed on chiffon fabrics. At the same time, this research is to provide a prediction model of the dominant color determination for double-sided heterochromatic digital printing. The dominant color model has a wide range of applications. It can help the designers to understand when heterochromatic color pairs are mixed on the front and back, and the color rendered will be closer to whichever color, i.e., which color is more dominant. The prediction models of the range of color differences created under subjective and objective measurements are offered *via* the research of subjective and objective experimental data. Through the combination usage of the objective color difference model and the subjective color difference model, people can understand whether the printing effect of the designed color is acceptable to the human eye during the double-sided digital printing process, so as to improve the color matching problem of double-sided digital printing with heterochromatic colors. This model is suitable for translucent natural fibers printed with activated fuels, and the key conclusions of this research are as follows.

#### From the objective experiment, the following conclusions can be derived

(1) Based on the analysis of color difference intensity of the front and back of double-sided digital heterochromatic printing, it can be seen that color performance tends to be closer to the lighter color in double-sided heterochromatic digital printing. So, in the actual production, one needs to choose a pair of colors with similar lightness for double-sided digital printing. The prediction model for the dominant color determination is as follows:

*Dominant color determination*


(1) $$\begin{array}{l} △E^{*}{}_{\left(\;f-b\right)}=-0.102+4.554△L^{*}{}_{\;(f-b)}-1.084△C^{*}{}_{ab(\;f-b)}\\ \;+0.024△H^{*}{}_{\;ab(f-b)}(R=\;0.745) \end{array}$$

(When ΔE^*^_f − b_ < 0, the positive color is the dominant color)

(2) The objective color difference of double-sided heterochromatic digital printing is closely related to the lightness and chroma of the color and shows a negative correlation; the objective color difference range of double-sided heterochromatic digital printing is affected by the lightness difference between single- and double-sided digital printing and the chroma difference, which is the most affected by the lightness difference. The prediction model of the objective color difference range is as follows.

*Objective color difference range*


(2) $$\begin{array}{l} △E^{*}{}_{\left(d-s\right)}=\;5.884-0.698△L^{*}{}_{\left(d-s\right)}-0.114△C^{*}{}_{ab(d-s)}\\ \;+\;0.497△H_{ab(d-s)}^{*}(R=\;0.854)\; \end{array}$$

#### From the subjective measurement experiments, the following conclusions can be derived

(1) The perceived color difference in double-sided heterochromatic digital printing is negatively related to the lightness and chroma of the color. The lightness difference between single- and double-sided digital printing influences the range of perceived color difference in double-sided heterochromatic digital printing. The color difference range prediction model is as follows.

*Subjective prediction of composite visual score*


$$\begin{array}{l} C\;=\;1.956-0.135△L^{*}{}_{\left(d-s\right)}-0.006△C^{*}{}_{ab(d-s)}\\ \;+\;0.119△H^{*}{}_{ab(d-s)}(R=0.743)\;(6) \end{array}$$

(2) When men and women make subjective assessments of experimental samples, female observers are more color tolerant than males, but the fitting degree of the regression equation of the color difference range between men and women shows that female observers' experimental results are closer to the objective results than shown by male observers.
(3) According to the correlation model between L^*^, a^*^, b^*^ and subjective visual score for experimental samples with subjective measurement < 3, when doing double-sided heterochromatic digital printing, the color with higher lightness (55–75) in the first quadrant and chromaticity value a^*^b^*^ between 15 and 55 need to be chosen. Colors in the second quadrant with lightness values between 20–65 and smaller b^*^ value (0–15) can be matched in pairs with colors in the fourth quadrant with lightness values between 30–45 and smaller a^*^b^*^ values (10–25).

#### Given the objective experiment and subjective measurement experiment, the following conclusions can be drawn

The law of color presentation under subjective measurement is closely connected to the law of color presentation under objective measurement (Pearson correlation coefficient is 0.84). When selecting colors for double-sided heterochromatic digital printing, lightness has the greatest impact on color difference, followed by the hue difference.

### Prospects

By combining subjective assessment of psychophysics experiment and objective instrument measurement, the effects of color lightness, chroma, and color relative to color rendering were studied during double-sided heterochromatic digital printing. The associated prediction model is also provided. In terms of color selection, the chosen colors are unevenly distributed in CIELAB space, and the colors in the third quadrant are absent, and therefore the sample size of the selected colors may be increased in future trials. Because the subjective experiment is an observation of the combined effect of perceived lightness, chroma, and hue, the experimental results are closely related to the subjects' own color learning ability and the experimental conditions, and the experiment conducted in a dark room included only subjects from university, with no experimental samples from other age groups. Alternative backdrop circumstances and the inclusion of additional age groups in future tests need to be attempted. This experiment was limited to translucent chiffon fabric, which is thinner and has a greater permeability than other silk fabrics, and the color rendering impact was influenced by the higher permeability in the chiffon fabric sample. In future studies, alternative colors can be tested to see if the pattern design has an effect on the color rendering. With the ongoing growth of color technology and the expansion in human life's desire for color in apparel, the research of the color presentation pattern for double-sided heterochromatic digital printing of textiles has a broad range of possibilities.

## Meaning of symbols

In the paper, all terms of the dominant color model, objective color difference model, and subjective color difference model are shown in Supplementary Table 3.

## Data availability statement

The original contributions presented in the study are included in the article/Supplementary material, further inquiries can be directed to the corresponding authors.

## Ethics statement

Ethical review and approval was not required for the study on human participants in accordance with the local legislation and institutional requirements. The patients/participants provided their written informed consent to participate in this study. Written informed consent was obtained from the individual(s) for the publication of any potentially identifiable images or data included in this article.

## Author contributions

MZ and YD contributed to the conception and design of the study, performed the statistical analysis, and wrote the manuscript. MZ and HY performed the experimental part. All authors contributed to the revision, read, and approved the submitted version.

## Funding

This work was supported by the Youth Project of Zhejiang Provincial Natural Science Foundation (No. LQ19C090009), and the Research Fund of Zhejiang Sci Tech University (Nos: 19012201-Y, 18012108-Y, and XMJWCb20200029). The authors declare that this study received funding from Honghua Digital Technology Co., Ltd. The funder was not involved in the study design, collection, analysis, interpretation of data, the writing of this article, or the decision to submit it for publication.

## Conflict of interest

Authors HZ and LX are currently employed by Hangzhou Honghua Digital Technology Co., Ltd. Author YD is doing postdoctoral cooperation research in Hangzhou Honghua Digital Technology Co., Ltd. The remaining authors declare that the research was conducted in the absence of any commercial or financial relationships that could be construed as a potential conflict of interest. The reviewer PR declared a shared affiliation with the author YD to the handling editor at the time of review.

## Publisher's note

All claims expressed in this article are solely those of the authors and do not necessarily represent those of their affiliated organizations, or those of the publisher, the editors and the reviewers. Any product that may be evaluated in this article, or claim that may be made by its manufacturer, is not guaranteed or endorsed by the publisher.
