# Supplementary material for: Exploring the law of color presentation of double-sided heterochromatic digital printing for textile
Source: Front Psychol. 2022 Oct 18;13:956748. doi: 10.3389/fpsyg.2022.956748 (PMC9624189; doi:10.3389/fpsyg.2022.956748)
Supplement: Supplementary file 1 [file Table_1.docx]

**Supplementary Material**

**TABLE 1** Chroma value L*a*b* of 15 colors

| **Design color (standard color card)** | | | | **Actual color (5-layer test method)** | | | |
| --- | --- | --- | --- | --- | --- | --- | --- |
| Color preview | L* | a* | b* | Color preview | L* | a* | b* |
|  | 57.4 | 70.8 | 44.8 |  | 57.7 | 47.9 | 24.7 |
|  | 68.0 | 48.8 | 23.0 |  | 70.7 | 32.1 | 7.4 |
|  | 83.1 | 23.1 | 9.0 |  | 81.1 | 16.2 | -1.2 |
|  | 97.3 | -20.1 | 84.1 |  | 86.4 | -10.2 | 58.3 |
|  | 97.8 | -16.4 | 60.4 |  | 87.4 | -9.5 | 39.9 |
|  | 98.7 | -9.7 | 30.7 |  | 89.1 | -2.6 | 10.7 |
|  | 79.3 | 13.3 | -31.5 |  | 79.6 | 4.9 | -17.2 |
|  | 94.5 | -31.9 | 24.3 |  | 85.7 | -11.5 | 8.3 |
|  | 67.1 | 43.1 | 74.0 |  | 65.6 | 40.5 | 48.0 |
|  | 73.8 | 28.6 | 61.9 |  | 72.8 | 23.1 | 38.7 |
|  | 81.9 | 15.9 | 41.1 |  | 81.1 | 12.5 | 17.2 |
|  | 90.7 | 6.4 | 19.8 |  | 85.3 | 7.5 | 5.4 |
|  | 32.0 | 51.6 | -32.9 |  | 46.5 | 26.2 | -25.5 |
|  | 37.4 | 37.0 | -24.5 |  | 49.6 | 28.6 | -23.0 |
|  | 45.0 | 18.8 | -12.8 |  | 56.9 | 18.4 | -15.4 |
