# Supplementary material for: Exploring the law of color presentation of double-sided heterochromatic digital printing for textile
Source: Front Psychol. 2022 Oct 18;13:956748. doi: 10.3389/fpsyg.2022.956748 (PMC9624189; doi:10.3389/fpsyg.2022.956748)
Supplement: Supplementary file 2 [file Table_2.docx]

**Supplementary Material**

**TABLE 2** L*a*b* values of 6 colors

| **Color Preview** | **L*** | **a*** | **b*** |
| --- | --- | --- | --- |
|  | 53.24 | 80.09 | 67.20 |
|  | 87.74 | -86.18 | 83.18 |
|  | 32.30 | 79.19 | -107.87 |
|  | 62.53 | -8.68 | -46.30 |
|  | 93.53 | -14.84 | 91.85 |
|  | 49.53 | 78.71 | -3.78 |
