# Supplementary material for: Exploring the law of color presentation of double-sided heterochromatic digital printing for textile
Source: Front Psychol. 2022 Oct 18;13:956748. doi: 10.3389/fpsyg.2022.956748 (PMC9624189; doi:10.3389/fpsyg.2022.956748)
Supplement: Supplementary file 3 [file Table_3.docx]

**Supplementary Material**

**TABLE 3** Meaning of Symbols

| **Symbol** | **Meaning of Symbols** |
| --- | --- |
| △E* _f_ /△E* _b_ | The color difference between the front / back of the fabric and the standard sample |
| L*_ab (f)_/ L* _ab(b)_ | Lightness value on the front / back of the fabric |
| C*_ab(f)_ /C*_ab(b)_ | Chroma value on the front / back of the fabric |
| h_f_ / h _b_ | Hue value on the front / back of the fabric |
| △E* _f-b_ | △E*_f_－△E* _b_ |
| △L* _f-b_ | L*_f_－L* _b_ |
| △C*_ab (f-b)_ | C*_f_－C* _b_ |
| △H*_ab(f-b)_ | △H*_ab(f-b)_ = [(△E* _ab_) ² －(△L* _f-b_) ² －(△C* _f-b_) ²]^1/2^ |
| L* _b_ / L* _b_ | Lightness value on the double / single of the back |
| C*_ab(b)_ /C*_ab(s)_ | Chroma value on the double / single of the back |
| h _d_ / h _s_ | Hue value on the double / single of the fabric |
| △E* _d-s_ | Color difference between double-sided and single-sided samples from DE2000 |
| △L* _d-s_ | L*_d_－L* _s_ |
| △C*_ab (d-s)_ | C*_d_－C* s |
| △H*_ab(d-s)_ | △H*_ab(d-s)_ = [ (△E* _ab_) ² －(△L* _d-s_) ² －(△C* _d-s_) ²]^1/2^ |
| F | Subjective prediction of female visual score |
| M | Subjective prediction of male visual score |
| C | Subjective prediction of composite visual score |
